# Editorial: Next generation therapeutic modality to cure autoimmune diseases

**DOI:** 10.3389/fimmu.2025.1631995

**Published:** 2025-06-17

**Authors:** Guobao Chen, Ce Wang, Qi Wan, Feng Dong, Timothy Radstake

**Affiliations:** Cambridge Research Center, AbbVie Inc, Cambridge, MA, United States

**Keywords:** next generation therapeutic modality, hematopoietic stem cell transplant (HSCT), CAR T cell therapy, Treg-based cell therapy, high-throughput medicinal chemistry (HTMC), cures, B cell-mediated autoimmune diseases, CD19 CAR T cell therapy

Autoimmune diseases pose a major medical challenge as the immune system erroneously targets the body’s own tissues, resulting in persistent inflammation and significant health consequences. The traditional treatment regime generally suppresses the immune system broadly, offering only symptomatic relief and increasing susceptibility to infections. However, recent strides in understanding the immune system coupled with breakthroughs in technology have sparked a new wave of treatments aimed at specific pathogenic pathways to restore balance and potentially offer a long-lasting cure without compromising overall immune function. Recent advancements in technologies such as stem cell therapies, chimeric antigen receptor (CAR) T cell therapies, targeted protein degradation approaches, and nucleotide-based therapies, hold the promise of drug-free remission and potential cures by focusing precisely on pathogenic cells without overall immune suppression.

To encourage more researchers to work towards novel therapies that could potentially bring cures to autoimmune disease patients, a Research Topic on *Next Generation Therapeutic Modality to Cure Autoimmune Diseases* was proposed and hosted by a group of AbbVie scientists working on novel modalities and joined by a few co-editors from other organizations in 2024/2025. This Research Topic has attracted many submissions on different novel drug modalities and resulted in 11 successful publications covering topics from novel small molecule drugs to different types of cell therapies, that are revolutionizing the therapeutic options for patients and challenging the status quo of standard care.

Although small molecule drugs are considered as traditional drug modalities, new class of small molecule modulators are still emerging as novel immune system regimens to reach immune balance in a milder way. There are 6 publications focusing on novel small molecule modulator approaches, covering from novel small molecule discovery platform to novel classes, combinations and novel analytic methods to connect drugs with new indications. The novel small molecule discovery platform designed by Dada et al., the Sulfur-Fluoride Exchange (SuFEx) click chemistry-based high-throughput medicinal chemistry (HTMC) platform used a “Direct-to-Biology” approach to generate a focused library of tamoxifen analogs and screened them in a cell-based pseudo-Ebola virus infection assay, that greatly shortened the timeline for drug discovery. Li et al. have examined the therapeutic potential of coumarins, a novel class of aromatic natural products capable of modulating immune cells and regulating inflammatory cytokines. This research highlights coumarins’ promise in treating autoimmune diseases like type 1 diabetes, ulcerative colitis, systemic lupus erythematosus, rheumatoid arthritis, and multiple sclerosis by affecting key signaling pathways. This review provides the insight into the connection between coumarins and autoimmune diseases and enable the discovery of effective and safe drugs for autoimmune diseases. Similarly, Jonić et al. reported their discovery of a new class of fluorescent aryl hydrocarbon receptor (AHR) ligands, AGT-5, that promoted a general immunosuppressive environment in their mouse model in the pancreas and small intestine lamina propria at the early phase of disease, and thereby inhibit the severity of Type 1 diabetes (T1D) in mice. On the other hand, Zhang et al., reported the potential expansion of abrocitinib, an oral small-molecule Janus kinase 1 (JAK1) inhibitor, to be used for Lichen amyloidosis (LA) associated with severe atopic dermatitis (AD). A novel combination of low-dose cyclophosphamide with Chinese herbal medicine Shuli Fenxiao formula was reported by Du et al., for the treatment of intermediate-to-high risk primary membranous nephropathy (PMN), which provided an efficacious and safe option for intermediate-to-high risk PMN patients, particularly elderly patients with contraindications to corticosteroid use or those with refractory disease. Lastly, Fu et al. applied drug-target Mendelian Randomization (MR) to study IL-6 receptor inhibitor effects and utilized novel analytic methods to explore the therapeutic potential across multiple diseases.

While cell therapies were not initially a primary focus for treating autoimmune diseases, recent advancements have led to increased interest and research in this area with 5 publications recruited in this Research Topic. Traditionally, autoimmune diseases have been managed with immunosuppressive medications and other conventional treatments. However, cell therapies, which involve using living cells to modulate the immune system or regenerate damaged tissues, are gaining more tractions as potential options for treating autoimmune conditions. Research is ongoing to explore their effectiveness and safety in this context. Bode et al. reported a new therapeutic option: using genetically engineered beta cells to shape autoimmunity, giving valuable insights for future therapeutic advancements to treat and cure Type 1 diabetes (T1D). Arve-Butler and Moorman provided a comprehensive examination of tolerogenic adjuvants currently utilized in tolerogenic vaccines, which induce antigen-specific tolerance by promoting tolerogenic antigen presenting cells, regulatory T cells, and regulatory B cells, and/or by suppressing/depleting antigen-specific pathogenic T and B cells. In addition, Wong et al., Liang et al. and Bulliard et al. further illustrated the potential of novel cell therapies to cure autoimmune diseases.

Hematopoietic stem cell transplant (HSCT) therapies are emerging as a rescue approach for patients in whom many other immunomodulatory therapies are not successful. Wong et al. reported a detailed clinical and electrophysiological response to HSCT in a patient with autoimmune retinopathy (AIR). After undergoing HSCT, symptoms of photopsia rapidly discontinued, evidenced by improvement of retinal function and visual field recovery via objective electroretinography and optical coherence tomography. Additionally, a 22-month follow-up demonstrated a sustained clinical response. This case report highlights the novelty that HSCT may work as a therapeutic method for selected patients of refractory AIR that failed to respond to traditional immunotherapy.

Although cell therapies are emerging to address the unmet needs that are not covered by traditional therapies, such as small molecule immune suppressants and biologics, there are critical differences between different types of cell therapies. Liang et al. summarized the recent advances in cell therapies and delineated the differences between stem cell transplantation and CAR T therapy. The authors listed the advantages of targeted cell therapy over stem cell therapy on specific elimination of pathogenic cells directly in disease tissue to achieve complete immune reset, exemplified by the success of CD19 CAR T therapies in the clinic on achieving complete immune reset in lupus and a few other B-cell driven autoimmune diseases. The chimeric autoantibody receptor (CAAR) T cell approaches were also discussed, which could potentially deplete only specific autoantibody producing cells, including B cells and plasma cells. The authors evaluated the status of CAR T therapies against different target cells and different disease types and listed the potential and limitations of these therapies for immune mediated inflammatory diseases.

With the latest advancements in the rapidly evolving cell therapies, Bulliard et al. further summarized and discussed current innovative advancements in regulatory T cell (Treg)-based cell therapies in offering targeted and durable disease remission for autoimmune and immune mediated diseases. In addition to highlighting the groundbreaking advances in CD19 CAR T cell therapy for B cell-mediated autoimmune diseases, the authors explore the therapeutic potential of Type 1 and Foxp3+ CAR Treg cells. These strategies aim to restore immune homeostasis locally and softly, minimizing adverse effects associated with generalized immunosuppression. This review emphasizes the transformative potential of cutting-edge therapeutic strategies to redefine treatment paradigms, address critical unmet needs, and advance toward curing autoimmune diseases.

In summary, emerging novel drug modalities and innovative therapies are leading the revolution of the treatment regimens for autoimmune diseases. These innovative therapies hold the promise of drug-free remission and potential cures by focusing precisely on pathogenic cells or specific mechanisms without overall immune suppression. With the advancements in novel technologies, the standard of care will be advancing more and more towards the cures for all autoimmune diseases.

